# Macrophage Activation in Acute Exacerbation of Idiopathic Pulmonary Fibrosis

**DOI:** 10.1371/journal.pone.0116775

**Published:** 2015-01-15

**Authors:** Jonas Christian Schupp, Harald Binder, Benedikt Jäger, Giuseppe Cillis, Gernot Zissel, Joachim Müller-Quernheim, Antje Prasse

**Affiliations:** 1 Department of Pneumology, University Medical Centre, Freiburg, Germany; 2 Institute of Medical Biostatistics, Epidemiology and Informatics, University Medical Center, Johannes Gutenberg University, Mainz, Germany; 3 Faculty of Biology, University of Freiburg, Freiburg, Germany; 4 Respiratory Diseases Section, Department of Clinical Medicine and Immunological Sciences, University of Siena, Siena, Italy; French National Centre for Scientific Research, FRANCE

## Abstract

**Background:**

Acute exacerbation (*AE*) of idiopathic pulmonary fibrosis (IPF) is a common cause of disease acceleration in IPF and has a major impact on mortality. The role of macrophage activation in AE of IPF has never been addressed before.

**Methods:**

We evaluated BAL cell cytokine profiles and BAL differential cell counts in 71 IPF patients w/wo AE and in 20 healthy volunteers. Twelve patients suffered from AE at initial diagnosis while sixteen patients developed AE in the 24 months of follow-up. The levels of IL-1ra, CCL2, CCL17, CCL18, CCL22, TNF-α, IL-1β, CXCL1 and IL-8 spontaneously produced by BAL-cells were analysed by ELISA.

**Results:**

In patients with AE, the percentage of BAL neutrophils was significantly increased compared to stable patients. We found an increase in the production rate of the pro-inflammatory cytokines CXCL1 and IL-8 combined with an increase in all tested M2 cytokines by BAL-cells. An increase in CCL18 levels and neutrophil counts during AE was observed in BAL cells from patients from whom serial lavages were obtained. Furthermore, high baseline levels of CCL18 production by BAL cells were significantly predictive for the development of future AE.

**Conclusions:**

BAL cell cytokine production levels at acute exacerbation show up-regulation of pro-inflammatory as well as anti-inflammatory/ M2 cytokines. Our data suggest that AE in IPF is not an incidental event but rather driven by cellular mechanisms including M2 macrophage activation.

## Introduction

Idiopathic pulmonary fibrosis (IPF) is a fatal disease with limited treatment options [1]. Although IPF has an overall poor prognosis, it is now recognized that its clinical course varies from slow progression to acute exacerbation with subsequent respiratory deterioration and death [2–4]. Independent of the status of pulmonary function, an accelerated progression of respiratory symptoms and deterioration of pulmonary function may occur at any stage in the course of the disease. These episodes are called ‘‘acute exacerbations’’ (AE) of IPF [5]. Martinez and colleagues [4] reported that in their IPF cohort 47% of deaths followed an acute deterioration in respiratory symptoms. Recently, two studies [6, 7] reported the 1-year incidence of AE as 8.5% and 14.2%, respectively. Song and colleagues [6] also showed that AE was the most common cause of rapid deterioration in IPF. After the initial diagnosis, the median survival of patients with AE was much shorter than that of patients without any episode of rapid deterioration. Thus, AE is thought to be an important factor affecting mortality in IPF.

The mechanisms and causes of AE in IPF are poorly understood and have only partially been studied so far [8]. Currently, it is debated whether AE is an externally induced, incidental event or a result of underlying cellular mechanisms [9]. Lung pathology of IPF patients with AE is very similar to that of acute respiratory distress syndrome (ARDS) including diffuse alveolar damage and hyaline membranes [10, 11]. Hence, a diffuse injury to alveolar epithelial cells was postulated. Gene expression studies of lung tissues indicated that AE of IPF is characterized by enhanced epithelial injury and proliferation as compared to stable IPF; reflected by increases in Cyclin A2, alpha-defensins and apoptosis of epithelium [12].

Fibrotic lung diseases including IPF are associated with a distinct type of macrophage activation called M2 or alternative activation [13, 14]. Classical/M1 macrophage activation by microbial agents and/or Th1 cytokines, in particular by interferon gamma (IFN-γ), induces the production of interleukin 12 (IL-12). On the other hand, macrophages stimulated by Th2 cytokines disclose a different activation pathway called alternative activation or M2 which plays a critical role in tumour progression and wound healing [15]. A profibrotic role of alternatively-activated alveolar macrophages in IPF was demonstrated in humans [16] as well as in mouse models [13, 17–19]. We reported recently, that collagen induces a profibrotic M2 type of alveolar macrophages [20] via CCL18 [16]. Based on these findings, we became interested in investigating the activation type of alveolar macrophages from IPF patients with acute exacerbation. Therefore, a comprehensive panel of chemokines produced by classically (IL-1β, TNF-α, CXCL1, IL-8) and by alternatively (CCL2, CCL17, CCL18, CCL22, IL-1ra) activated macrophages [21] was evaluated.

## Material and Methods

### Subjects

Over a period of seven years, seventy-one consecutive, therapy-naive patients with IPF, who were administered to our tertiary referral centre to undergo a standardized bronchoscopy with bronchoalveolar lavage (BAL) (as previously described [22, 23]) during routine diagnostic work-up, and twenty healthy volunteers were included in the study after obtaining their written informed consent. The patients were diagnosed according to the consensus statement criteria by clinical evaluation, high resolution computed tomography, histologic and laboratory findings [2]. Patients with underlying collagen vascular disease, occupational diseases or other identifiable causes of usual interstitial pneumonitis were excluded. Acute exacerbation in IPF was defined as a sudden aggravation of dyspnea within 30 days, in which any other identifiable causes have been excluded and new ground glass opacity and/or consolidation in HR-CT have been documented [3]. BAL microscopy, microbiological cultures and PCR for various viruses were used to rule out infectious disease. In seven patients two consecutive BALs were obtained, one at initial diagnosis and a second BAL later during AE. All patients were followed up in our outpatient clinic, seen at least every three months, instructed to visit the outpatient clinic immediately if acute respiratory distress occurred and were monitored for the development of acute exacerbations. Five patients had undergone lung transplantation and were censored for event-free survival analysis. Neither the IPF patients nor the healthy donors currently smoked at the time point of BAL. The treatment regimens varied, some patients were treated with prednisone, azathioprine, cyclophosphamide and N-acetylcysteine. The varying treatment regimens hindered us to evaluate a treatment effect on development of AEs. The study was approved by the local ethics committee of Albert-Ludwig University Freiburg (231/03).

### Bronchsocopy and BAL procedure

Patients received local anaesthesia (oxybuprocaine) and midazolam as needed. After intubation, the bronchoscope was placed in wedge position in the middle lobe and 300ml of per-warmed saline was installed by aliquots of 20ml. Directly after installation of each 20ml aliquot BAL was harvested, pooled and placed on ice. None fraction was discarded.

### BAL cell isolation and culture

BAL cells isolation and culture were immediately performed after bronchoscopy as previously described [24]. Cell differentials were determined using cytosmear and May-Grünwald-Giemsa staining counting of at least 300 cells. Cells were >90% viable by Trypan blue exclusion. Cells were resuspended and cultured in RPMI-1640 (Gibco) with 2% heat-inactivated, human AB serum with antibiotics (50 U/ml penicillin and 50 µg/ml streptomycin, Biochrom, Germany) in 24-well plastic plates (1×10^6^ cells/ml/well) in a humidified atmosphere containing 5% CO_2_ at 37ºC for 24 h. Cell-free supernatants were kept at-80ºC for later analysis.

### ELISA

IL-1ra, chemokine (C-C motif) ligand 2 (CCL2), CCL17, CCL18, CCL22, CXCL1, TNF-α, IL-1β and IL-8 were quantified using DuoSet ELISA Development System Kits (R&D Systems Europe, UK) according to the manufacturer’s protocol. All samples were measured in duplicate, for duplicate samples an intra-assay coefficient of variation (CV) of < 10% and inter-assay CV of < 20% were accepted.

### Statistical analysis

Values are expressed as mean ± SD and a statistical significance level of 0.05 was used. To compare patients’ characteristics we used the Mann–Whitney U test or Fisher’s exact test, as appropriate. The Wilcoxon signed-rank test was used to compare paired values before and during an AE. Cox proportional hazards models were used to examine the effect of the risk for AE for patients without AE at baseline. For adjustment, we considered baseline FVC percent predicted, age, gender, and body weight and height. We first considered each of these candidates for adjustment in the univariate Cox model and used only those significant at a level of 5% for multivariate analysis. Results were summarized as hazard ratios, representing the relative risk of developing an AE as a result of a specific characteristic during the entire period of observation. For markers found to be significant, the median value was used as a cut-off to obtain two risk groups with corresponding Kaplan-Meier estimates and 95% confidence intervals.

## Results

### Patient characteristics

Seventy-one patients with IPF and twenty healthy volunteers were studied. The healthy volunteers were slightly, yet significantly, younger, had a similar gender distribution, a similar percentage of positive smoking history and a normal pulmonary function test. At baseline twelve patients suffered from an AE (17%). During the 24-month follow-up period sixteen patients suffered from an AE of their IPF. Altogether 26 patients died during follow up, of these, 15 patients died in the context of AE (58%). Patients with AE were statistically significantly younger and had worse FVC, DLCO and composite physiologic index (CPI) [25] values compared to IPF patients without AE (Table 1). Healthy volunteers had a significantly lower percentage of neutrophil and eosinophil granulocytes and a significantly higher percentage of lymphocytes of BAL cells then patients with IPF. Furthermore, BAL cytology showed a significantly higher percentage of neutrophil granulocytes and a significantly lower percentage of lymphocytes and alveolar macrophages in patients with AE (Table 1).

**Table 1:** Demographic characteristics and spontaneous production of macrophage derived chemokines of patients with or without acute exacerbation.

	**Controls n = 20**	**IPF without AE at BAL n = 59**	**IPF with AE at BAL n = 12**	**p value IPF vs AE**
**Age [years]**	60.9 ± 8.5*	68.2 ± 8.9	62.9 ± 8.0	p = 0.03
**Sex [male/female]**	16/4	47/12	12/0	n.s., p = 0.19
**Ex-smokers [%]**	75.0	67.3	72.7	n.s., p = 0.72
**Disease duration [months]**	not applicable	18.2 ± 28.7	23.4 ± 19.2	n.s., p = 0.06
**Baseline FVC [%]**	105.6 ± 18.4^†^	67.2 ± 18.8	52.3 ± 20.6^‡^	p = 0.047
**Baseline FEV1 [%]**	96.4 ± 21.3^†^	66.2 ± 18.1	54.7 ± 18.4^‡^	n.s., p = 0.07
**Baseline DL_CO_ [%]**	not done	50.2 ± 17.9^#^	28.3 ± 2.4^‡^	p = 0.008
**CPI**	not applicable	61.2 ± 5.2^‡^	44.8 ± 13.3^‡^	p = 0.02
**IL-1ra [ng/ml]**	18.9 ± 14.8*	32.2 ± 38.1	72.6 ± 50.8	p = 0.003
**CCL2 [ng/ml]**	0.7 ± 1.6*	1.7 ± 4.1	3.7 ± 5.0	p = 0.002
**CCL17 [pg/ml]**	17.6 ± 12.0*	54.6 ± 78.9	77.2 ± 44.3	p = 0.03
**CCL18 [ng/ml]**	2.5 ± 1.8^†^	15.8 ± 18.9	25.0 ± 16.3	p = 0.02
**CCL22 [ng/ml]**	0.4 ± 0.3^†^	1.8 ± 1.8	3.5 ± 2.6	p = 0.009
**IL-8 [ng/ml]**	49.0 ± 37.8*	80.1 ± 52.0	126.1 ± 27.8	p = 0.02
**CXCL1 [ng/ml]**	1.5 ± 1.9	2.4 ± 5.1	16.5 ± 22.8	p = 0.01
**TNF-α [ng/ml]**	0.4 ± 0.4	0.6 ± 1.1	1.5 ± 1.7	n.s., p = 0.06
**IL-1β [ng/ml]**	0.1 ± 0.2*	0.2 ± 0.5	0.5 ± 0.8	n.s., p = 0.32
**Total cells/100 ml BAL [×10^6^]**	9.8 ± 5.1	12.0 ± 7.8	9.9 ± 5.4	n.s., p = 0.47
**AM [%]**	79.2 ± 12.5	74.0 ± 15.5	60.3 ± 17.7	p = 0.008
**LYM [%]**	16.2 ± 9.8*	11.6 ± 9.4	5.7 ± 4.4	p = 0.02
**PMN [%]**	2.0 ± 1.4^†^	9.4 ± 10.2	27.3 ± 19.2	p < 0.0001
**EOS [%]**	0.5 ± 0.6^†^	4.5 ± 5.4	6.2 ± 4.7	n.s., p = 0.10

**Definition of abbreviations:** AE: patients with an acute exacerbation at baseline; FVC: forced vital capacity; DLco: diffusing lung capacity for carbon monoxide, CPI: composite physiologic index, AM: alveolar macrophage; LYM: lymphocyte; PMN: polymorphonuclear neutrophils; EOS: eosinophils; n.s.: not significant. Data are expressed as mean ± standard deviation.

*p value < 0.05 and

^†^p value < 0.0001 for comparison of controls and all patients with IPF; p values comparing IPF patients w/o AE are listed in the last column.

^‡^Not feasible for all patients.

### Spontaneous cytokine production of BAL cells

**Figure 1:**
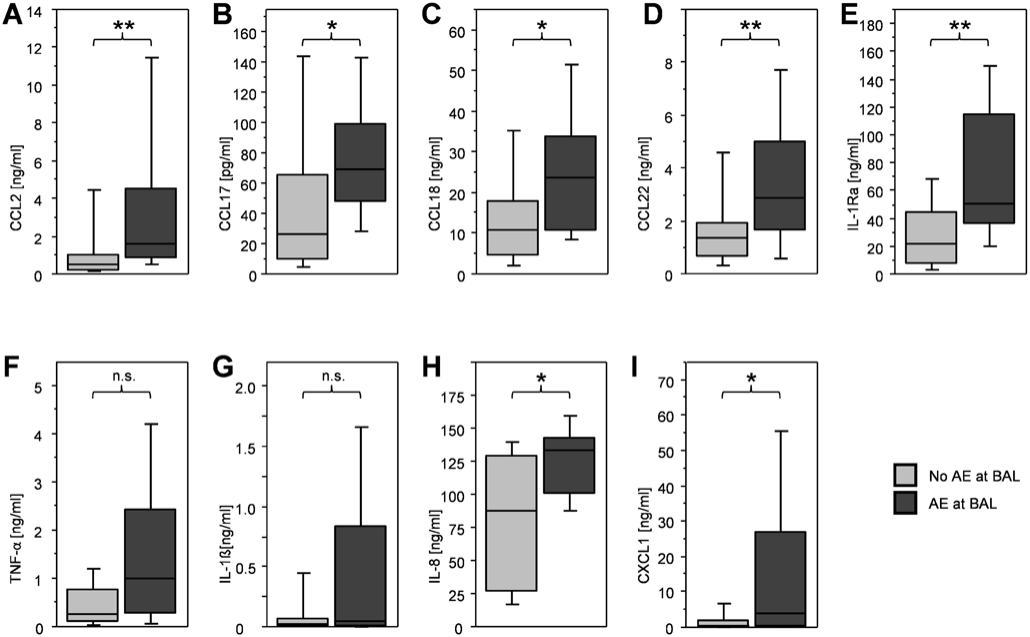
Spontaneous macrophage derived chemokine production in IPF patients w/o acute exacerbation. Boxplots of the spontaneous production of A: CCL2, B: CCL17, C: CCL18, D: CCL22, E: IL-1ra, F: TNF-α, G: IL-1β, H: IL-8 and I: CXCL1 protein by BAL cells of patients with IPF. The dark grey boxplots represent patients who suffered from an acute exacerbation (AE, n = 12), the light grey represent patients who did’t suffer from an AE at timepoint of BAL (NoAE, n = 59) (* p<0.05, ** p<0.01, n.s. = not significant).

BAL cells of patients with IPF produced significantly more CCL2, CCL17, CCL18, CCL22, IL-1ra, IL-1β and IL-8 than cells of healthy volunteers (Table 1). There was no difference between IPF patients and controls in TNF-α and CXCL1 production. We compared the spontaneous production of macrophage derived cytokines of the patients with IPF who suffered from an acute exacerbation at time point of BAL against those patients who did not. The twelve patients with acute exacerbation had significantly higher levels of CCL2 (p = 0.002), CCL17 (p = 0.03), CCL18 (p = 0.02), CCL22 (p = 0.009), IL-1ra (p = 0.003), CXCL1 (p = 0.01) and IL-8 (p = 0.02) (Table 1 and Fig. 1). TNF-α and IL-1β were not significantly elevated, although TNF-α showed a trend towards statistical significance. Of the tested chemokines, only CCL2 correlated weakly with baseline FVC % predicted (r = -0.4, p = 0.001) and with baseline CPI (Speamans rho = -0.46, p = 0.003).

### Increase of CCL18 and neutrophils during AE in serial BAL measurements

**Figure 2:**
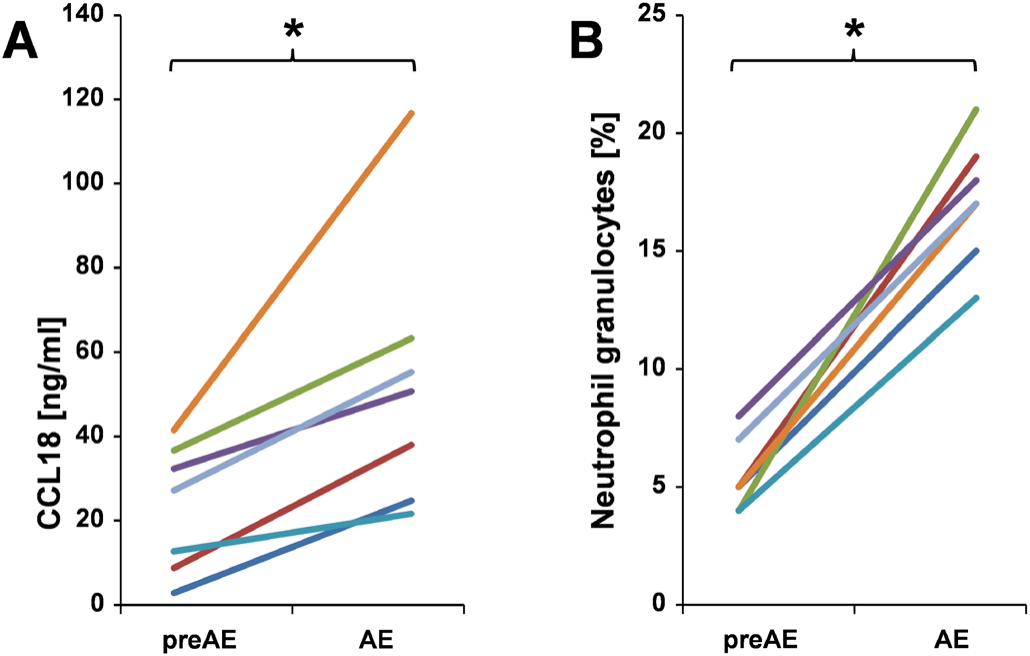
Course of spontaneous production of CCL18 and neutrophil cell count before and during an AE. A: Course of spontaneous production of CCL18 protein by BAL cells and B: Percentage of neutrophile granulocytes of BAL of seven patients with IPF before (preAE) and during an AE (AE). Each color represents a distinct patient. (* p<0.05).

From seven patients with IPF we obtained BAL at baseline and during AE at a later time point. The spontaneous chemokine production at initial diagnosis and during the acute exacerbation was measured (Fig. 2). CCL18 production was significantly increased (p = 0.04) during acute exacerbation. CCL18 was elevated despite a non-significant decrease of alveolar macrophages from 79.8% to 69.8% on average and a significant increase of neutrophils from 5.8% to 18.0% on average (p = 0.04) during acute exacerbation in these patients. There was a non-significant trend towards increased levels of the M2 chemokines CCL17, CCL22, and IL-1β during AE (data not shown).

### Spontaneous production of CCL18 by BAL cells predicts risk for AE

Univariate Cox models were used to quantify the risk for developing an AE for IPF patients without AE at baseline. The variates tested were as follows: all measured cytokines and patient characteristics like baseline FVC percent predicted, age, gender, body weight and height. Of the tested demographic characteristics only baseline FVC (percent predicted) reached statistical significance, therefore baseline FVC (%) was the only parameter included in the multivariate analysis (Table 2). The high spontaneous production of both CCL18 (p = 0.0009) and IL-8 (p = 0.049) had a significant effect in univariate Cox models (Table 2).

**Table 2:** Estimated effects from univariate Cox regression models for acute exacerbations.

	**coefficient**	**hazard ratio [confidence interval]**	**p**
**Age [years]**	-0.012	0.98 [0.94—1.04]	n.s., p = 0.55
**Sex (male = 1)**	-0.50	0.61 [0.14—2.72]	n.s., p = 0.51
**Body height [m]**	3.06	21.27 [0.02—> 100]	n.s., p = 0.34
**Body weight [kg]**	0.013	1.013 [0.97—1.07]	n.s., p = 0.58
**Baseline FVC [%]**	-0.036	0.965 [0.94—1.00]	p = 0.02
**CCL2 [ng/ml]**	0.050	1.05 [0.98—1.13]	n.s., p = 0.17
**CCL17 [pg/ml]**	0.0002	1.00 [0.99 -1.01]	n.s., p = 0.96
**CCL18 [ng/ml]**	0.030	1.031 [1.02—1.04]	p = 0.0009
**CCL22 [ng/ml]**	0.15	1.16 [0.95—1.40]	n.s., p = 0.13
**IL-1ra [ng/ml]**	0.0036	1.004 [0.99 -1.02]	n.s., p = 0.53
**CXCL1 [ng/ml]**	0.0002	1.00 [1.00 -1.00]	n.s., p = 0.71
**IL-8 [ng/ml]**	0.011	1.011 [1.00—1.02]	p = 0.049
**TNF-α [pg/ml]**	-0.0003	1.00 [1.00 -1.00]	n.s., p = 0.40
**IL-1β [pg/ml]**	-0.0004	1.00 [1.00 -1.00]	n.s., p = 0.58

**Definition of abbreviations:** FVC: forced vital capacity; n.s.: not significant.

**Figure 3:**
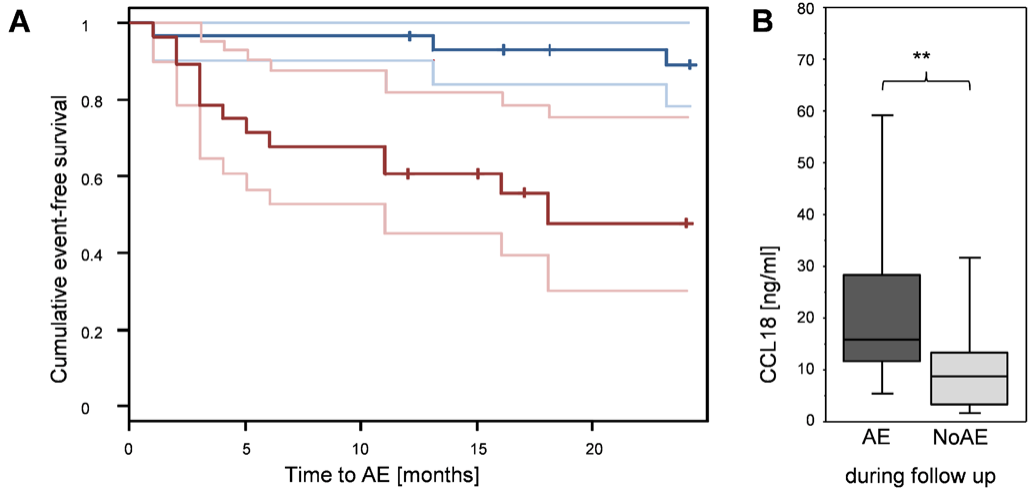
Risk for acute exacerbation in IPF patients is dependent on spontaneous CCL18 production. A: Kaplan-Meier curves of the 59 followed-up IPF patients without AE at baseline with acute exacerbation as outcome event for two risk groups, obtained by splitting at the median CCL18 concentration (median = 10.8 ng/ml), including 95% confidence intervals (light lines). The red lines represent the group of IPF patients with a spontaneous CCL18 production levels above the median; the blue line represents the group of IPF patients with a spontaneous production levels below the median. B: Spontaneous production of CCL18 by BAL cells of IPF patients with no AE at the time point of BAL. The dark grey boxplots depict CCL18 levels of patients who developed AE during follow up, while in light grey CCL18 levels are shown of patients who never suffered from AE (NoAE) (** p<0.01).

In the multivariate analysis only CCL18 showed a significant effect after adjusting for the effect of FVC (p = 0.002). Note this effect is still significant when adjusting for multiple testing. The spontaneous production of IL-1ra, CCL2, CCL17, CCL22, TNF-α and IL-1β did not have a significant effect on event-free survival. The hazard ratio of CCL18 was similar with or without adjusting for FVC at baseline in the multivariate or the univariate Cox proportional hazard model, respectively (HR = 1.032 per ng/ml vs. HR = 1.031 per ng/ml). When defining two risk groups by splitting at the median CCL18 concentration of 10.8 ng/ml (median absolute deviation = 6.4 ng/ml), the Kaplan-Meier curves and corresponding 95% confidence intervals of the two groups differed considerable in event rates (Fig. 3A). Patients without AE at initial diagnosis, who developed AE during follow up had a significantly higher spontaneous production of CCL18 compared to patients who never had an AE (Fig. 3B).

## Discussion

Acute exacerbation of IPF is major factor contributing to IPF mortality. Exogenous as well endogenous factors were described to trigger acute exacerbation [12, 26, 27], but underlying pathomechanisms are poorly understood. Histology of acute exacerbation in IPF resembles ARDS [11, 28], however BAL findings have never been reported systematically so far. The results of this study demonstrate up-regulation of cells and cytokines, which were previously shown to be increased in the context of acute lung injury. Moreover, cytokines associated with wound healing processes were also increased in acute exacerbation of IPF. Taken together, our data suggest that underlying endogenous mechanism related to macrophage activation highly influence the patients risk to develop acute exacerbation of IPF.

In line with Kurosu et al. [29], the percentage of neutrophils in BAL was significantly increased in IPF patients at the time of AE compared to more stable patients. Furthermore, percentage of lymphocytes and macrophages were significantly reduced at acute exacerbation of IPF while percentage of eosinophils was unchanged. This cellular pattern of acute exacerbation of IPF is very similar to BAL cell differentials of ARDS, yet with lower neutrophil cell counts. Thus, consistent with histology findings, BAL cell differentials of acute exacerbation of IPF resemble ARDS and indicate acute lung injury [30].

We expected to find many pro-inflammatory cytokines known to be involved in acute lung injury to be up-regulated in acute exacerbation of IPF. We found, however, only distinct pro-inflammatory cytokines significantly up-regulated such as IL-8 and CXCL1 while the increase in TNF-alpha and IL-1beta levels failed to reach statistical significance. IL-8 and CXCL1 are both potent neutrophil chemoattractants and paralleled lung injury and neutrophil sequestration in mice models of ARDS [31, 32]. Of interest, also in ARDS TNF-alpha levels are not significantly up-regulated, while a pivotal role for IL-1beta in ARDS was documented [33, 34]. There may be several reasons as to why an increase in spontaneous IL-1beta production during AE was not as evident as expected in our study. First, BAL was obtained during acute exacerbation, but not necessarily at the time point of initiation. Thus, we may have missed the peak of IL-1beta production at the very beginning. Furthermore, IL-1β is tightly regulated and its basal production rate is very low. Noteworthy in the context of our study is that all tested pro-inflammatory cytokines are mainly macrophage derived and markers of the classical type of macrophage activation (M1). This is the first study demonstrating increased spontaneous production of distinct pro-inflammatory cytokines by BAL-cells during acute exacerbation of IPF.

In acute lung injury with up-regulation of proinflammatory M1 cytokines we expected to find down-regulation of cytokines which indicate alternative macrophage activation. However, there was a striking increase in all tested M2 cytokines such as IL-1ra, CCL2, CCL17, CCL18 and CCL22 at AE of IPF. Moreover, in patients with serial lavages, at baseline and during acute exacerbation, we also found a significant increase of the M2 cytokine CCL18. There was a trend of increased M2 cytokines but this did not reach statistical significance because of the low patient number with serial lavages. Thus, in patients with acute exacerbation the macrophage activation type is shifted further towards alternative activation. The serial analysis of cytokines before and during an AE is limited due to a very small study population, so these results have to be confirmed in a bigger cohort. Taken together, our data indicate a specific type of macrophage activation occurs in acute exacerbation of IPF which consists of an up-regulation of pro-inflammatory (M1) cytokines as well as cytokines associated with M2.

Our study was not aimed at investigating why there is an increase in M2 production during acute exacerbation. However, we noted that many of the M2 cytokines up-regulated in AE are induced by IL-1β signalling such as CCL2, CCL22 and IL-1ra [35, 36]. Acute lung injury and increase in IL-1β levels may contribute to the observed shift towards M2 activation. In mice, overexpression of IL-1β induces acute lung injury and leads to chronic fibrosis [37, 38]. Furthermore, it was reported that injury to alveolar epithelial cells induces M2 macrophage activation [39] and macrophage dependent fibrosis. M2-activated macrophages have been shown to promote wound healing including attraction of neutrophils and various progenitor cells that close the wound. In IPF these repair processes seem to fail which results in an ongoing wound healing response and persistent M2 activation. Our data indicate that processes of acute lung injury as well as of wound healing are highly up-regulated during acute exacerbation of IPF.

Increased production of CCL18 by BAL-cells in patients with IPF was associated with an increased risk to suffer from acute exacerbation in the course of the disease. The effect was independent of other variables like gender, age or baseline lung function. Of interest, although increased IL-8 and CCL18 serum levels had already been shown to be prognostic for survival prediction in IPF, these cytokines had not yet been linked to an increased risk for acute exacerbation in IPF. The finding that increased production of CCL18 by macrophages is associated with a high risk for acute exacerbation was unexpected and clearly argues against acute exacerbation being an incidental event triggered by exogenous factors. Our data show that there is a considerable heterogeneity in cytokine production at initial diagnosis of IPF. Some patients already have high M2 cytokine production early in their disease, while others have low M2 cytokine levels even in advanced disease. Thus, our data suggest that some patients are prone to acute exacerbations while others are not and the risk for acute exacerbation is, at least partly, reflected by M2 cytokine production levels.

The underlying mechanisms as to why baseline levels of M2 activation are related to the evolution of AE in IPF are unclear. M2 activation in IPF is considered to be present in the context of a wound healing response and mislead ongoing repair processes. It can be speculated that baseline M2 levels correlate with the amount of damage and wound. The level of alveolar epithelial damage and wound at baseline reflected by CCL18 production may be related to the fragility of alveolar epithelial cells and thereby may indicate the risk to develop severe acute lung injury in response to trigger factors.

## Conclusions

Our data demonstrate a neutrophil influx and a distinct type of macrophage activation with features of M1 as well as M2 in acute exacerbation of IPF. Moreover, the study provides evidence for an endogenous, macrophage-driven mechanism in acute exacerbation of IPF, which influences on the patient risk to develop acute exacerbations. Acute exacerbation of IPF appears to be not an incidental event solely driven by exogenous trigger factors rather a condition evoked by disease underlying pathomechanisms. These pathomechanisms which are already present in stable IPF appear to be exaggerated during acute exacerbation. On this background, it may be worth exploring modulation of macrophage activity as a potential therapeutic approach to prevent acute exacerbations of IPF.
